# The association of positive educational experiences with adolescents’ depressive symptoms: longitudinal evidence from a trial cohort in English secondary schools

**DOI:** 10.3389/fpubh.2026.1860546

**Published:** 2026-07-16

**Authors:** G. J. Melendez-Torres, Veena Muraleetharan, Gemma Lewis, Ruth Ponsford, Rebecca Meiksin, Maria Lohan, Neisha Sundaram, Honor Young, Rona Campbell, Chris Bonell

**Affiliations:** 1 Department of Public Health and Sport Sciences, University of Exeter, Exeter, United Kingdom; 2 Department of Public Health, Environments and Society, London School of Hygiene & Tropical Medicine, London, United Kingdom; 3 Division of Psychiatry, Faculty of Brain Sciences, University College London, London, United Kingdom; 4 Department of Medicine, University of Pittsburgh, Pittsburgh, PA, United States; 5 Medical Biology Centre, School of Nursing and Midwifery, Belfast, United Kingdom; 6 Hitotsubashi Institute for Advanced Study, Hitotsubashi University, Kunitachi, Tokyo, Japan; 7 DECIPHer, Cardiff University, Cardiff, United Kingdom; 8 Bristol Medical School, Bristol, United Kingdom

**Keywords:** adolescent, education, longitudinal research, mental health, school

## Abstract

**Introduction:**

The school environment influences adolescent health through both individual experiences, such as one’s own engagement with school, and broader school-level factors, such as aggregate student levels of engagement, affecting all students. For mental-health outcomes, existing evidence more consistently supports individual-level influences than school-level effects. In relation to depression, negative student-level educational experiences have been associated with increased symptoms, while evidence for school-level associations remains limited and often methodologically weak. We aimed to address this gap through a secondary analysis of longitudinal data from a trial involving 49 English secondary schools.

**Methods:**

Student-reported educational experiences (school belonging, educational commitment, participation at school, relationships with teachers and expectation of university) as well as family affluence were measured at baseline (ages 12–13) and depressive symptoms were assessed at follow-up (ages 15–16). Associations were examined at both the individual student and aggregate school level of educational experiences.

**Results:**

Forty-five schools were retained at endline and 4,601 students comprised our analytical sample. At the student level, greater school belonging (beta coefficient = −0.24; 95% confidence interval = −0.29, −0.19) and educational commitment (beta coefficient = −0.19; 95% confidence interval = −0.29, −0.08) were associated with lower risk of depressive symptoms in fully adjusted models. At the school level, only overall educational commitment was associated with reduced depressive symptoms in a partially but not fully adjusted model (beta coefficient = −1.02; 95% confidence interval = −1.86, −0.18).

**Discussion:**

These findings suggest that educational experiences influence depressive symptoms primarily through individual-level mechanisms, for example involving students’ sense of belonging, engagement, participation and relationships) but the general level of educational commitment within a school may also play a meaningful role. (249 words).

## Introduction

Adolescence is a critical period for the onset of mental-health problems, with around two-thirds of all mental-health disorders manifesting by age 25 (1). Across many countries including the UK, young people’s mental health has significantly worsened since the 1980s particularly among girls (2). These declines are more dramatic than declines among adults and have centred on ‘internalising’ problems, such as depression and anxiety more than ‘externalising’ problems such as misbehaviour and aggression (3). Poor mental health in adolescence is associated with mental ill-health in adulthood (4), and can also cause more immediate harms during adolescence; depressed or anxious students are more likely to be absent from school or stop attending altogether (5, 6), and students with poor mental health or wellbeing are less likely to do well in academic exams (7–9).

In the UK, mental-health support teams have been set up to mainstream mental-health promotion in schools but there are concerns that these are not delivering effective interventions (10). Studies suggest that school mental-health promotion interventions have inconsistent effects (11–14) and there are concerns that lessons on mental health and some early interventions might worsen mental health by increasing distress or labelling everyday challenges as mental-health problems thus exacerbating avoidance and anxiety (15, 16). Whole-school interventions might offer a means of promoting mental health by building more positive school climates, and building a positive sense of engagement and sense of school belonging among students while avoiding any harmful mechanisms (14). While there is some evidence that such interventions can improve various mental-health outcomes (17, 18), evidence is limited (19). Observational studies could make a useful contribution by identifying what positive aspects of school environments might be protective against adverse mental-health outcomes, which whole-school interventions could then address.

There is evidence from observational studies that children and young people’s mental health might be affected by their experiences at school and the schools they attend. At the individual level, there is strong evidence that mental health is better among students with certain school experiences. More school connectedness or belonging is associated with reduced risk of depression, anxiety and distress (20–25). Good relationships with peers and with teachers, and happiness in school are all associated with reduced risk of depression (21). Feeling safe at school is associated with reduced risk of depression and other mental-health symptoms (21, 24). Lower school stress or worry is associated with reduced risk of distress (20, 21). It is also well established that family affluence is associated with reduced risk of depression among adolescents (26).

There is also some evidence that school-level factors can influence student mental health over and above individual educational experiences. However, while school-level factors significantly explain variance in mental-health outcomes, they account for less of this than they do for aggression and substance use (24, 27–29), and less than the variance explained by individual-level factors (21, 30, 31). Nonetheless, some school-level factors are significantly associated with better mental-health outcomes. A large cohort study tracking students aged 11–14 in UK secondary schools found lower risk of depression, fewer social, emotional and behavioural difficulties, and higher well-being were all associated with both individual students’ own positive perceptions of school climate and the overall school-level average of these perceptions (28). Students attending schools with more positively rated climates tended to have better mental-health outcomes, even after accounting for their own individual perceptions of the school environment. Similarly, a large cohort study of English primary schools found that higher school-level average ratings of student-reported positive school climate were associated with fewer emotional and behavioural symptoms and reduced odds of mental-health difficulties (32).

However, beyond such general measures of overall school climate, we are aware of only one study that has examined how more specific school-level factors are associated with mental-health outcomes, which relied on cross-sectional study designs, limiting ability to draw causal inferences. This study in English primary schools found that school-level measures of the quality of provision for personal development and well-being, as well as overall levels of academic achievement, were associated with reduced student risk of psychological difficulties (33).

This paper aims to address this research gap by using longitudinal evidence to examine what specific aspects of school environments as reported by individual adolescents at age 12–13 years and as aggregated at the school level are associated at with subsequent student-reported depressive symptoms when the adolescents are aged 15–16 years. We also examined whether these associations vary by sex, which is plausible given both adolescent mental health and school experiences differ by sex (21). The exposures examined were derived from student baseline reports of sense of school belonging (whether they feel they ‘fit in’ at school), commitment to education (whether they feel committed to learning), active participation at school (whether they get involved in broader school life), good relationships with teachers, expectations of attending university (as well as family affluence). The last of these is not a measure of educational experience but it still does describe the social environment of the school. Our hypothesis was that all these factors are associated with reduced risk of depression but more strongly at the individual than the school level.

## Methods

### Study design and participants

This study is a secondary analysis of longitudinal data collected within the Positive Choices open cohort two-arm cluster randomized controlled trial (34). This trial was publicly registered (ISRCTN16723909). It was guided by a protocol which includes a power calculation for the sample size (Supplementary material 1). Forty-nine schools were recruited from counties in central and south England (Supplementary material 2). Eligible schools were of any type except those only for students excluded from mainstream schools or with special educational needs and disabilities, or judged ‘inadequate’ by government school inspectors. They were recruited via emails.

We conducted baseline student surveys (December 2021–March 2022) before randomisation and endline surveys around 33 months later. At baseline, we obtained enrolment lists of students in year 8 (aged 12–13) during the 2021/22 school year. Students deemed competent by schools to consent were eligible. At endline, students enrolled in year 11 (aged 15/16) during the 2024/25 school year were eligible. Students completed paper questionnaires in classrooms. Fieldworkers offered support. Teachers remained at classroom fronts, not viewing student responses. Informed consent was obtained from headteachers for trial participation, and students and parents for data collection. Students and parents received information 1 week before surveys and could, respectively, opt out themselves or their child. Immediately before the survey, students received verbal and written information, and could ask questions before deciding whether to opt in. London School of Hygiene & Tropical Medicine research ethics committee (reference 26,411) approved the study.

### Measures

All measures are described in Supplementary material 3. Exposures were assessed based on student reports. Sense of school belonging, educational commitment, school participation and relationships with teachers were assessed at baseline using subscales of the Beyond Blue School Climate Questionnaire, respectively scored 1–32, 1–16, 1–24 and 1–40 with higher scores indicating more positive experiences (35). Expectations of attending university were assessed at baseline using the Ripple binary measure of expecting to be at university at age 20 (36). Family affluence was assessed at baseline using the Family Affluence Scale, focusing on family ownership and consumption (37) with total scores ranging 0–13 and higher scores indicating more affluence. Regarding covariates, students were asked at baseline about their sex, ethnicity, sexual orientation and family structure, while data on school characteristics (sex intake, national school inspectorate rating, number of students and attainment in public 16+ examinations) were taken from government school performance tables. Regarding outcomes, depressive symptoms were assessed at endline using the eight-item Patient Health Questionnaire (PHQ-8) (38), a modified version of the nine-item PHQ-9 (39). Total scores ranged 0–24 with higher scores indicating more severe symptoms. No other mental-health outcomes were collected.

### Analysis

In the trial, there was poor fidelity of delivery of key intervention components and no differences between arms in primary or second outcomes. Additional information can be found in the trial protocol (Supplementary material 1) and main trial paper (34). Hence, we decided that our sample for the present analysis could draw on all trial participants, adjusting for arm. Complete case analysis was utilised to deal with the low levels of missing data.

First, our analysis aimed to describe the characteristics of the schools and students, as well as our exposures and levels of depressive symptoms, using simple counts and percentages. Next, we looked at how students’ depressive symptoms at the end of the study were associated with baseline measures of sense of school belonging, educational commitment, participation in school, relationships with teachers and expectations of going to university, as well as family affluence. These were analysed in terms of student-level measures and school-level averages. To understand whether the school environment had a contextual effect beyond individual experiences, we created a school-level average for each exposure and a student-level score centred within each school indicating how each student compared to others in their own school). We then examined whether school-wide levels of the exposures were more associated with reduced depressive symptomatology than would be expected from student-level measures alone, to assess whether there was a school-level association over and above student-level associations.

We estimated beta coefficients for associations using multilevel linear regression models accounting for clustering within schools, including a random intercept for schools. For each exposure, we ran three models. Model 1 included only the exposure variables (school-level average and student-level centred score), adjusting for trial arm. We also tested whether the school-level and student-level associations were significantly different from each other to assess contextual effects. Model 2 added school characteristics (sex intake, national school inspectorate rating, number of students and attainment in public 16+ examinations) and student characteristics (sex, ethnicity, sexual orientation and family structure) as covariates. This model was used to assess whether each exposure was associated with depressive symptoms independently of these other factors. Model 3 included all exposures and all covariates in the same model. This was done to allow a full interpretation of how all variables related to each other. For school-level factors, we anticipated that this model might over-adjust for variables that might lie on the pathway between the exposure and the outcome and so it was important to interpret the results of all models together when weighing evidence for associations. We did not adjust for depressive symptoms at baselines because students had already been in their schools for over a year so that baseline depressive symptoms may already have been influenced by school factors meaning that adjusting for baseline symptoms might remove part of the effect we aimed to assess. Finally, we re-ran model 2 to assess whether the associations differed between males and females. Because these were exploratory analyses that doubled the number of significance tests, we only report sex differences where the interaction was statistically significant at a reduced *p* value of *p* < 0.025 to reduce the chance of false positive findings (40).

As a final sensitivity analysis, we generated 10 multiple imputations with chained equations using all variables. We re-estimated analyses in Model 2 and compared these to unimputed results. We did not undertake a drop-out analysis given the open cohort design.

## Results

Out of 48 schools recruited, 45 were retained at endline. The response rates across both arms was 77.3% (6,970 students of 9,017 eligible) at baseline and 77.9% at endline (6,268 of 8,045 eligible) 33 months after baseline (Supplementary material 4). Overall, 4,601 students (73.4% of endline respondents) were successfully matched to baseline responses, reflecting turnover in school enrolment and potentially a small number of students for whom name changes on enrolment lists were missed. The number of complete cases are reported for each analysis.

Participating schools were broadly representative of English secondary schools (Supplementary material 5). School and student characteristics at baseline are reported in Tables 1 and 2 respectively. Mean student age was 12.5 years. Half of students were female and half male. The mean school belonging score was 15.1 out of a possible maximum of 24. For educational commitment, this was 10.0 out of 12. For school participation, this was 13.3 out of 18. For relationships with teachers, this was 20.1 out of 30. Just over 75% expected to go to university. Mean family affluence was 8.1 out of a possible 13. Most students (82.0%) identified as straight/heterosexual with 2.5% identifying as gay or lesbian, and 5.7% as bisexual. Just over three-quarters (76.8%) identified as White with 7.4% identifying as Asian/Asian British, 6.6% as Black/African/Caribbean/Black British and 6.9% as of mixed/multiple ethnicity. Just over three-quarters (75.3%) lived with two biological parents, 7.7% with a parent and step-parent and 16.3% with one parent. At endline, the mean score for depression was 7.2 out of a possible 24. A model including only a term for trial arm indicated that 2% (95% CI: 0.01, 0.04) of the variance in depressive symptoms was attributable to differences between schools with 98% attributable to differences between students.

**Table 1:** School characteristics at baseline.

School characteristics		Prevalence or score *N* (%) or mean (SD)
School sex mix^1^	Schools providing data	49
	Mixed	43 (87.8)
	Female only	4 (8.2)
	Male only	2 (4.1)
School type^1^	Schools providing data	49
	Voluntary-aided	4 (8.2)
	Voluntary-controlled	1 (2.0)
	Community	8 (16.3)
	Academy (converter mainstream)	20 (40.8)
	Academy (sponsor led)	9 (18.4)
	Foundation	3 (6.1)
	Free school	2 (4.1)
	Private	2 (4.1)
Faith schools^1^	Schools providing data	49
	Faith schools	6 (12.2)
	Non-faith schools	43 (87.8)
Ofsted rating^1^^,^ ^2^	Schools providing data	47
	Outstanding	7 (14.2)
	Good	31 (63.3)
	Requires improvement	4 (8.2)
	Not available	5 (10.2)
Attainment 8 measure of academic achievement (SD)^1^^,^ ^2^^,^ ^3^	Schools providing data	49
	Mean (SD)	47.2 (9.6)^4^
School size	Schools providing data	49
	Mean (SD)	1071.6 (428.8)

1 Data on school population, Ofsted ratings and school sex make up were taken from get-information-schools.service.gov.uk, accessed 27/01/2022.

2 Statistics for state-funded schools only.

3 Due to the Covid-19 pandemic, data from 2018 to 2019 were the latest available for Attainment 8 score and absence. These were taken from compare-school-performance.service.gov.uk/download-data, accessed on 25/01/2022 (absence data) and 27/02/2022 (Attainment 8 and Progress 8 scores).

4 Data for three schools were estimated using single regression imputation.

**Table 2:** Student characteristics at baseline and students’ depressive symptoms and endline.

Student characteristics and outcomes		*N* (%) or mean (SD)	Missing data
Age (years)		12.5 (0.5)	0.0
Female sex		2,274 (49.4)	0.0
School belonging (BBSQ subscale score 0–24)		15.1 (4.5)	0.7
Educational commitment score (BBSQ subscale score 0–12)		10.0 (2.0)	1.0
School participation (BBSQ subscale score 0–18)		13.3 (3.1)	1.3
Teacher relationships (BBSQ subscale score 0–30)		20.1 (6.0)	0.4
University expectations Y/N		3,191 (75.4)	8.0
Family affluence (family affluence score 0–13)		8.1 (2.2)	0.6
Sexual orientation	Straight or heterosexual	3,610 (82.0)	4.3
	Gay or lesbian	108 (2.5)	
	Bisexual	253 (5.7)	
	Unsure/questioning	249 (5.7)	
	Other	185 (4.2)	
Ethnicity	White	3,530 (76.8)	0.1
	Mixed/multiple ethnic groups	318 (6.9)	
	Asian/Asian British	342 (7.4)	
	Black/African/Caribbean/Black British	303 (6.6)	
	Other ethnic group	103 (2.2)	
Family structure	Two parents	3,415 (75.3)	1.5
	Parent plus step-parent	349 (7.7)	
	One parent	738 (16.3)	
	Other	31 (0.7)	
Endline depressive symptoms score (PHQ8) score 0–24 with higher scores indicating more symptoms		7.2 (6.1)	7.8

Regression models are presented in Table 3. In model 1, at the student level, higher levels of school belonging, educational commitment, school participation, teacher relationships and family affluence but not university expectations were associated with lower depressive symptoms. School-level estimates largely followed the same pattern as student estimates but were only statistically significant for school belonging, educational commitment and teacher relationships. Comparing school-level average and student-level score centred within each school for each exposure, we found no evidence of contextual effects in analyses, with the exception of educational commitment (*p* = 0.001). This contextual effect suggests there was a ‘benefit’ of lower depression symptoms for students in ‘high-commitment’ schools over and above their own educational commitment.

**Table 3:** Associations with depressive symptoms.

Exposure		Associations with depressive symptoms					
		Model 1^*^		Model 2^**^		Model 3^***^	
		Beta coefficient	Sample size	Beta coefficient	Sample size	Beta coefficient	Sample size
Student exposures	School belonging	**−0.38** **(−0.42, −0.34)**	4,220	**−0.29** **(−0.33, −0.25)**	3,993	**−0.24 (−0.29, −0.19)**	3,659
	Educational commitment	**−0.49** **(−0.58, −0.4)**	4,207	**−0.41** **(−0.5, −0.32)**	3,979	**−0.19** **(−0.29, −0.08)**	
	School participation	**−0.28** **(−0.34, −0.22)**	4,198	**−0.25** **(−0.3, −0.19)**	3,971	−0.02 (−0.1, 0.06)	
	Teacher relationships	**−0.19** **(−0.22, −0.16)**	4,230	**−0.16** **(−0.19, −0.12)**	4,001	−0.02 (−0.06, 0.03)	
	University expectation	−0.13 (−0.59, 0.32)	3,923	−0.37 (−0.81, 0.08)	3,717	0.11 (−0.34, 0.56)	
	Family affluence	**−0.27** **(−0.36, −0.17)**	4,220	**−0.17** **(−0.27, −0.08)**	3,989	**−0.15** **(−0.25, −0.06)**	
School exposures	School belonging	**−0.46** **(−0.74, −0.18)**	4,220	−0.16 (−0.47, 0.15)	3,993	0.03 (−0.63, 0.69)	
	Educational commitment	**−1.69** **(−2.41, −0.98)**	4,207	**−1.02** **(−1.86, −0.18)**	3,979	−1.31 (−2.86, 0.23)	
	School participation	−0.4 (−0.76, −0.05)	4,198	−0.11 (−0.47, 0.24)	3,971	0.06 (−0.51, 0.62)	
	Teacher relationships	**−0.23** **(−0.38, −0.07)**	4,230	−0.13 (−0.29, 0.03)	4,001	0.01 (−0.48, 0.5)	
	University expectations	−0.62 (−3.75, 2.52)	3,923	−1.82 (−4.97, 1.33)	3,717	−1.22 (−5.15, 2.7)	
	Family affluence	−0.25 (−0.56, 0.07)	4,220	−0.05 (−0.38, 0.29)	3,989	0.02 (−0.4, 0.45)	
Intra-cluster correlation coefficient		0.012 (0.004, 0.034)					

All estimates are presented as beta (95% CI).

^*^Adjusted only for intervention group.

^**^Additionally adjusted for school and student characteristics.

^***^Adjusted for all variables. Bold values indicate statistical significance *P* < 0.05.

In model 2, including a vector of student-level and school-level confounders alongside each pair of student and school exposures did not change patterns of magnitude or significance for student-level exposures. However, at school level, only higher levels of average educational commitment remained associated with reduced depressive symptoms. No contextual effects were identified in these models. Multiply imputed analyses (see Supplementary material 6) did not generate meaningfully different results in any model. In model 3 analyses, only student-level school belonging, educational commitment and family affluence remained significant predictors, suggesting their continued association even when accounting for other closely related variables.

Only one sex-stratified analysis was statistically significant at *p* < 0.025. University expectations were significantly associated with reduced depressive symptomatology at the student level, but only for females (males *β* = 0.14, 95% CI [−0.44, 0.73]; females *β* = −1.04, 95% CI [−1.71, −0.37]; interaction *p* = 0.009).

## Discussion

### Summary of key findings

We found evidence that, for depressive symptoms, school-related exposures operating at the individual level appeared much more influential than at the school level. These results support similar findings from previous studies (21, 24, 27–31).

At the student level, there was evidence that school belonging, educational commitment, school participation and good relationships with teachers, as well as family affluence, were associated with reduced risk of depressive symptoms. This association remained even in fully adjusted analyses for school belonging, educational commitment and family affluence. These findings add longitudinal evidence to that from existing cross-sectional studies finding a protective effect of individual students’ sense of school belonging (20–25) and good relationships with teachers (21). Previous research has not examined associations between depression and expectations of attending university, educational commitment or active participation at school. Our study extends existing evidence by suggesting that individuals’ educational commitment might be a particularly important protective factor.

Our study makes an important novel contribution to the literature by examining what specific aspects of the school-level environment [rather than merely general ratings of overall school climate (32, 33)] are associated in longitudinal data with subsequent mental-health outcomes. We found that the only school-level factor that appeared important in its association with reduced depressive symptoms was educational commitment, where there was evidence that, in partially adjusted models, school-wide elevated levels of student educational commitment might be protective against depressive symptomatology over and above individual-level effects of this factor. This broadly aligns with the cross-sectional evidence cited earlier that, in English primary schools, higher school-level academic achievement is associated with lower student mental-health difficulties (33).

There was evidence of heterogeneity by sex for these influences only with regard to university expectations, which were significantly associated with reduced depressive symptoms at the student level for females but not males. It might be that high aspirations are more protective for girls than boys because sense of purpose is more important for girls or that high aspirations help protect girls against patriarchal or other iniquitous gender norms which harm mental health, although we know of no empirical evidence on these questions and indeed qualitative research suggests academic pressure can feel highly gendered for girls (41).

### Limitations

Although we report longitudinal associations, we cannot assume that these are causal since they might be subject to residual confounding from unmeasured factors. As noted, we did not adjust for baseline depressive symptoms because these could have lain on the causal path from exposures to outcomes given our focus was on year-8 students who had been at the school for some time. Our results might be confounded by depressive symptoms prior to entry to the school. Additionally, as mentioned in our methods, our models adjusting for all variables may have over-adjusted for factors lying on the causal pathway between exposures and outcomes so should be interpreted cautiously and alongside evidence from the other models.

Our evidence, particularly for student-level associations, is vulnerable to same-source bias (42) given our assessment of both exposures and outcomes relied on student reports. Future research might use measures derived from teacher assessments. Our findings of associations between lower depression symptoms and student-level reports of factors such as educational commitment and school belonging may also reveal more about underlying student dispositions than modifiable school processes.

It is possible that there are school-level mechanisms that more significantly influence young people’s mental health that we have failed to detect because these operate very similarly across all or most schools so not being liable to detection in studies comparing schools. There is a potential parallel here with school influences on physical activity at school, which does not vary very much between schools despite obviously being influenced by schools through school games lessons but in a similar way across schools (43). Finally, we cannot assume that our findings are generalisable beyond secondary schools in England.

### Implications for research and policy

Our findings, alongside previous studies, suggest that whole-school interventions that modify school environments and educational experiences might offer a promising means of promoting adolescent mental health, likely at lower cost than targeted interventions and avoiding possible iatrogenic effects (14–17). Our findings suggest that such interventions might most promisingly aim to build individual students’ sense of school belonging and commitment to learning. Such interventions might benefit individual students’ mental health but also achieve ‘herd-level’ benefits via increases in aggregate student educational commitment.

It is likely that some schools struggling with high levels of student needs and reduced capacity will struggle to implement multi-component whole-school interventions (44). It is possible that such schools can still build student educational commitment and school belonging by delivering a recently developed category of brief intervention targeting students and teachers (45–48). Such interventions involve, for example, a booklet providing students with survey results and stories from older students explaining how challenges and worries about school are normal and can improve with time, rather than indicating students do not belong in a school. Such interventions have been found to reduce disciplinary incidents and academic test anxiety, and improve sense of school belonging and educational attainment (45, 48). Similarly, brief teacher interventions aims to help teachers take an empathic approach to misbehaving students via online reading and reflection exercise. Trials have found evidence that these can reduce school suspensions (46, 47).

The effects of such brief interventions on mental health have not been examined. Our limitations section above acknowledges that the student-level associations that we and others have found between student reports on school experiences and lower depression symptoms could simply reflect underlying student dispositions rather than processes that can be modified. There is therefore equipoise about the potential effectiveness of brief interventions in reducing depressive symptoms and other adverse mental-health outcomes This indicates the need for investment in rigorous trials of the effectiveness of such interventions rather than their immediate scale-up. Our team is currently piloting such an intervention in English secondary schools to assess impacts on mental health and violence.

## Data Availability

The raw data supporting the conclusions of this article will be made available by the authors, without undue reservation.
